# Methylxanthine Content in Commonly Consumed Foods in Spain and Determination of Its Intake during Consumption

**DOI:** 10.3390/foods6120109

**Published:** 2017-12-04

**Authors:** Juan M. Sanchez

**Affiliations:** Chemistry Department, University of Girona, 17003-Girona, Spain; juanma.sanchez@udg.edu; Tel.: +34-636-569-984

**Keywords:** methylxanthine, caffeine, theobromine, tea, coffee, chocolate, soft-drink

## Abstract

Methylxanthines present psychostimulant effects. These compounds have low toxicity and their consumption at moderate levels presents some beneficial health effects, whereas some significant risk appears at high levels. Samples of common types of methylxanthine-containing beverages and foods consumed in Spain were analyzed to determine their content. Caffeine was the methylxanthine that was most found in the samples investigated. Instant coffees gave the highest caffeine percentage (18–44 mg·g^−1^). Green and scented teas were found to have a caffeine dry-weight content (8–26 mg·g^−1^) equivalent to ground coffees (13–23 mg·g^−1^), but black and pu-erh teas (18–30 mg·g^−1^) had a higher caffeine content. The evaluation of the most conventional methods for preparing espresso coffees showed that an espresso contains between 88–116 mg of caffeine. In the case of tea beverages, the amount of caffeine present was 2–3 times smaller than in espresso coffees. Energy drinks showed a similar caffeine content (80–106 mg) as espresso coffees. Chocolates had the lowest caffeine content. It has been found that none of the foods evaluated reach the recommended daily intake limit of 400 mg of caffeine with a single dose. This limit can be reached with 4–5 doses in the case of coffees and energy drinks. In the case of chocolates, the methylxanthine compound detected at large levels was theobromine, with amounts ranging from 4 to 10 mg·g^−1^ for dark chocolates.

## 1. Introduction

Caffeine (1,3,7-trimethylxanthine), theobromine (3,7-dimethylxanthine), and theophylline (1,3-dimethylxanthine) are the most well-known compounds of the family of methylxanthines and are naturally present in tea leaves, yerba mate, coffee beans, cocoa beans, kola nuts and guarana berries. Moreover, caffeine is probably the most broadly consumed central nervous system stimulant in the world [1,2,3].

Over the last few decades, different ingredients have been added to foods and beverages as functional ingredients by many manufacturers. The addition of synthetic additives to beverages is not new as caffeine has long been added to soft drinks, mainly in cola soft drinks as a substitute for kola nut extract. Manufacturers have always claimed that caffeine is added to these beverages as a flavoring agent, but some scientific evidence suggests that this claim may not be correct and any flavor effect of caffeine will be a function of its concentration in the drink [4,5,6]. Nowadays, one of the clearest examples of the addition of functional ingredients is the case of “energy drinks”, of which the market has grown exponentially since their commercial introduction at the end of the 20th century to the point where there are now hundreds of different brands with added caffeine contents ranging from 85 to 1200 mg/L [7]. Among other factors, this has resulted in an increased interest in the determination of biologically active constituents present in food and beverages. Among these, methylxanthines are of great interest as their consumption, mainly caffeine, is widespread around the world [8].

Methylxanthines are adsorbed in the gastrointestinal tract and can penetrate into the central nervous system, exerting psychostimulant actions, which are more evident in acute intake [9]. These compounds, and caffeine in particular, are adenosine receptor antagonists in the brain and enhance arousal, mood, and concentration levels [10,11,12]. The toxicity of methylxanthines in humans is relatively low and moderate consumption presents some health benefits [9,13,14,15,16]. High levels are required to produce undesirable adverse side effects, such as diuresis, cardiovascular and metabolic effects, bronchial relaxation and increased secretion of gastric acids [15,17].

Caffeine is usually the methylxanthine compound that is present at the highest levels in foods and beverages. Scientific evidence indicates that a moderate daily caffeine consumption is not associated with adverse health effects in healthy adult populations [1,18,19], although notable behavioral effects occur at low to moderate doses (50–300 mg) [20]. The European Food Safety Authority (EFSA) has established that caffeine intakes from all sources of up to 400 mg/day and single doses of 200 mg do not raise concerns for adults in the general population [21]. Although there are no specific recommendations in the USA, the U.S. Food and Drug Administration (FDA) states that the same level of 400 mg/day is not associated with adverse health effects [15]. In the case of pregnant women, the recommended dose is reduced to 200–300 mg/day as caffeine crosses the placenta and increases catecholamine levels, which might cause growth problems to the fetus [1,15,21,22]. For children, it has been recommended that the consumption of caffeine should be limited to <3 mg/kg body weight per day [15,21,23,24]. In general, it is recognized that a daily intake of caffeine of >500–600 mg represents a significant health risk and may therefore be regarded as abuse [1]. Taking into account the natural levels present in foods and beverages, toxic effects only tend to appear upon an excessive consumption of those foods with large amounts of caffeine, such as coffee and caffeine-enriched dietary supplements [25], or if combined with drugs of abuse [26,27,28].

The US-FDA includes caffeine within the list of substances that are generally recognized as safe (GRAS) when used in cola-type beverages [29], with tolerance at 0.02% (200 mg·L^−1^), but there are no limits on the caffeine that may be added to other foods or beverages. Therefore, no foods or beverages containing caffeine are required to list caffeine content in their labels, only drugs must list caffeine content [30]. Energy drink manufacturers classify their beverages as liquid dietary supplements because they contain herbs and other natural ingredients and, therefore, have different regulatory issues and there is no limit on the caffeine that can be added to dietary supplements. In the European Union, a beverage is considered to have a high caffeine content when it contains >150 mg/L of caffeine, with the exception of those based on coffee, tea or coffee or tea extract, and its label must indicate “high caffeine content” and express this content in mg per 100 mL [31]. This regulation requires energy drink manufacturers to label their caffeine content.

In the specific case of chocolate and other cocoa-containing foods, theobromine is the methylxanthine that is present at higher levels, usually 3–10 times more than caffeine [32,33]. Although theobromine is a more potent cardiac stimulant than caffeine [34] and is toxic for a variety of mammals such as dogs [35], pharmacological assays have demonstrated that in humans, theobromine is less active than caffeine, with single oral lethal dose 50 (LD_50_) in rodents about 10 times higher than for caffeine [9]. Baggot et al. [34] found that theobromine has differential effects on mood and behavior in a population of healthy young adults according to dose, but oral doses of around 1000 mg, well above normal intakes, were needed to observe negative effects. They suggested that the psychological effects of chocolate might be due to a combined interactive effect of caffeine and theobromine.

Accurate data of the methylxanthine content of foods and beverages is required in order to determine exposure to these compounds. In the case of commercial beverages, it is not especially problematic because most companies make this information available and the results obtained in different studies indicate that values are fairly consistent. The problem arises in the assessment of the methylxanthine content in tea, coffee and chocolate because the results can vary significantly depending on the origin of the crop and its processing and the way the extract is prepared. Most studies only focus on soft drinks, including energy drinks, and coffee and tea because different reports have indicated that >98% of all caffeine consumed came from these sources, with coffee being the main source in people older than 18 years and soft drinks in children and teenagers aged 2–17 years [15,36]. Chocolates and other cocoa-containing foods have received less attention since their contribution in terms of caffeine to the diet is small (<1.5%) [36].

The objective of the present study was to determine the content of the different methylxanthines in the most common types of beverages and foods consumed that are present in Spanish supermarkets, including commercial soft drinks, coffees, teas, and chocolates. The most conventional methods used for extracting methylxanthines from ground coffee and tea leaves have also been evaluated and discussed.

## 2. Materials and Methods

### 2.1. Reagents

Caffeine, theophyline and theobromine were from Sigma-Aldrich (Darmstadt, Germany). Methanol for HPLC was from Scharlab (Barcelona, Spain). Ultrapure Milli-Q water (Millipore Iberica, Barcelona, Spain) with a resistivity of 18.2 MΩ·cm (at 25 °C) was used. Glacial acetic acid and petroleum ether, both for analysis, were supplied by PanReac AppliChem (Barcelona, Spain). Two hundred mg ExtraBond C18 solid-phase extraction (SPE) cartridges were from Scharlab.

### 2.2. Samples

Samples of many common types of beverages and foods consumed nowadays were obtained from different local stores. The information on the label of each sample was checked to confirm that samples from the same commercial brand corresponded to different production batches. Each individual sample was analyzed in duplicate (foods) or triplicate (beverages) and the mean obtained was used as an individual data.

In the case of beverages, 121 samples were evaluated: 45 corresponded to different types of cola soft drinks from three commercial brands (20 regular colas, 10 diet colas, 10 zero colas and 5 caffeine-free colas), 23 samples were obtained from four brands of energy drinks, 22 were bottled or canned teas, and the other 31 samples were from commercial chocolate drinks.

In the case of foods, a total of 136 samples were evaluated: 63 commercial chocolates (7 were white chocolates, 10 milk chocolates, and 46 dark chocolates), 40 teas, 23 ground coffees, and 10 instant coffees (2 of which were decaffeinated). To evaluate the real intake of caffeine when consuming a coffee beverage, espresso coffees from four different coffee-bars were obtained and measured.

### 2.3. Sample Treatment

For soda and energy drinks, samples were degassed in an ultrasonic bath to eliminate carbon dioxide and then diluted with Milli-Q water before analyzing by HPLC. For chocolate foods, official method 980.14 of the AOAC was used [37]. In the case of tea leaves, a two-step extraction with hot water at 90 °C (10 min each extraction) was applied [38,39]. The same extraction procedure with hot water at 90 °C was used for ground coffee but with only one extraction, as indicated by the international standard ISO 20481:2008 [40]. For chocolate drinks, a clean-up step with solid-phase extraction was applied to remove lipophilic components of the beverages [41]. All samples were filtered through a 45 μm cellulose acetate syringe filter (Teknoroma, Barcelona, Spain) before injection in the HPLC. See Supplementary Materials for more specific details about each sample’s treatment, method validation and quality parameters.

### 2.4. Instrumental and Chromatographic Conditions

Chromatographic separations were performed with a SpectraSYSTEM (Thermo Scientific, Waltham, MA, USA) liquid chromatograph which was equipped with a vacuum membrane degasser (SCM1000), a gradient pump (P4000), an autosampler with a column oven and a Rheodyne Model 7725 injection valve (AS3000), and a diode array detector (PDAPlus). Twenty μL standards and samples were injected for each analysis. Detection was performed at 273 nm. The acquisition of chromatographic data was performed by means of Chrom Quest software (v. 5.0, Thermo Electron, Waltham, MA, USA). A 20 cm × 4.6 mm i.d. C18 5 μm column (Teknokroma, Barcelona, Spain) protected with the corresponding guard column was used for the separations.

A two-solvent gradient elution was performed at a flow rate of 1 mL/min. The solvents used were (A) Milli·Q water containing 0.1% acetic acid (pH ≈ 3.2) and (B) methanol. The mobile phase composition started at 10% B for 4 min, increased linearly to 25% B in 1 min and held for 10 min, followed by a linear increase of B to 90% in 7 min. The final conditions were held for 8 min. For the cleaning and regeneration of the column between runs, a linear decrease of B to 10% in 5 min was applied and these conditions were held for 3 min. A total run time of 30 min plus 8 min of regeneration was required for each analysis.

### 2.5. Statistical Analysis

Statistical analyses were performed using SPSS for Windows, version 15.0 (SPSS Inc., Chicago, IL, USA). For calculations of statistical significance, two-sided testing was used and *p* < 0.05 was considered as significant. One-factor analysis of variance (ANOVA) was applied for data comparison between multiple groups and the Tukey post-hoc test was applied to determine the relationships between the groups. The Student’s *t*-test was applied when only two groups were compared.

## 3. Results

### 3.1. Analysis of Soda and Energy Drinks

**Figure 1:**
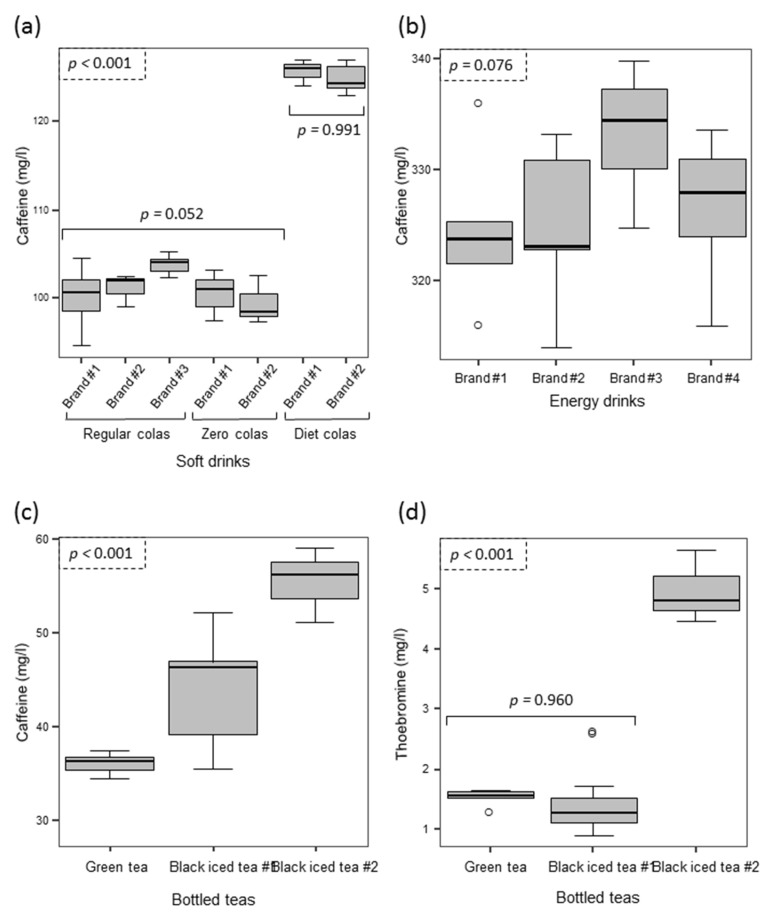
Box-plot displaying the distribution of the concentrations (in mg/L) detected in the samples analyzed for the different brands of soft drinks (**a**), energy drinks (**b**) and bottled teas for caffeine (**c**) and for theobromine (**d**).

A total of 90 beverage samples were evaluated: 45 samples corresponded to cola soda beverages (five of which were marketed as caffeine-free), 23 to energy drinks, and 22 to bottled or canned teas. The results obtained for the different type of beverages evaluated are shown in Figure 1.

The only methylxanthine detected in colas was caffeine, but this compound was never detected in the case of caffeine-free samples. For the other 40 cola samples, there was a significant difference in the caffeine content between the three different types of colas evaluated (i.e., regular, zero and diet colas, *p* < 0.001, Figure 1a). The post-hoc method used to determine the groups with the equivalent levels of caffeine showed the formation of two groups: the first was composed by the three brands of regular colas evaluated and the two brands of zero colas (*p* = 0.052), whereas the two brands of diet colas analyzed gave higher and equivalent caffeine levels (*p* = 0.991).

A second group of beverages evaluated consisted of energy drinks; 23 samples from four different brands with the same declared content of caffeine (320 mg/L) were evaluated. The results obtained indicate that there were no significant differences in their caffeine content (*p* = 0.076, Figure 1b). When the results obtained for each commercial brand were evaluated against the declared value, three of the brands did not yield any significant difference, whereas one of them (brand #3) yielded a significantly higher content than declared (*t*_calc_ = 6.66, *t_n_*_=7,α=0.05_ = 2.45).

The last group was bottled teas, where caffeine and theobromine were quantified in all samples; 22 samples from one green tea and two black iced tea brands were assessed. Significant differences were found in the caffeine content (*p* < 0.001, Figure 1c), with the lowest content being detected for the green tea beverage. In the case of theobromine, there were also significant differences between the three brands evaluated (*p* < 0.001), but brand #1 (green tea) and #2 (black iced tea) gave equivalent levels of theobromine (*p* = 0.960, Figure 1d).

### 3.2. Chocolates and Chocolate Drinks

Sixty-three samples from different commercial chocolate brands were tested. Theobromine and caffeine were the methylxanthines that were detected in all chocolate samples. Seven samples were white chocolates, which according to European Union (EU) legislation [42] must not contain cocoa solids and must contain >20% of cocoa butter. Ten samples corresponded to milk chocolates, which must contain no less than 25% of dry cocoa solids [42], for which the manufacturers declare a minimum content of 30%. The other 46 samples corresponded to dark chocolates (not less than 35% of total dry cocoa [42]), which were separated into four groups: <50% cocoa (*n* = 5), between 50% and 60% (*n* = 12), 70–75% (*n* = 18), and 80–85% (*n* = 11).

**Figure 2:**
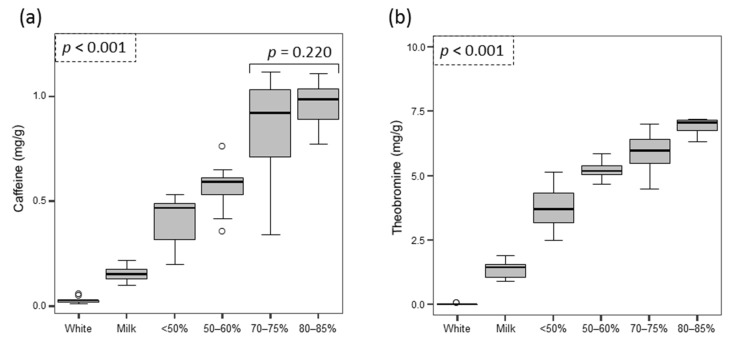
Box-plot displaying the distribution of the concentrations (in mg/g) of caffeine (**a**) and theobromine (**b**) detected in the different types of chocolate foods analyzed.

The content of both caffeine and theobromine showed significant differences between the different types of chocolates evaluated (*p* < 0.001, Figure 2) and for both compounds their content increased as the percentage of cocoa increased, as expected. In the case of caffeine, only the two types of chocolates with the largest amount of cocoa (70–75% and 80–85%) showed levels with no significant differences (*p* = 0.220), whereas all groups were significantly different according to their theobromine levels.

Thirty-one samples of chocolate drinks from 11 commercial brands were also evaluated. A mean caffeine level of 18.4 mg/L (standard deviation (sd) = 5.4) (median = 16.9 (14.9–21.5)) and a mean theobromine level of 205.7 mg/L (sd = 76.6) (median = 192.9 (156.3–266.1)) were found. The reported levels of cocoa content for the different commercial brands evaluated varied from 0.9% to 1.5%. Therefore, five new samples of the commercial brand with the lowest reported cocoa amount (0.9%) and seven new samples of brand with the highest amount of cocoa (1.5%) were analyzed and compared. A significant difference was obtained for the levels of caffeine content (*p* = 0.024), which were 13.3 mg/L (sd = 1.5) for the brand with 0.9% cocoa and 23.2 mg/L (sd = 7.2) for the brand with 1.5% cocoa, and for the theobromine content (*p* = 0.007), which were 148.4 mg/L (sd = 13.7) and 267.2 mg/L (sd = 79.6), respectively.

### 3.3. Tea Leaves

Forty different commercial teas were evaluated, analyzing three different samples for each tea. The teas were classified into four different groups by the information provided by the manufacturers: pu-erh (*n* = 10), black (*n* = 11), green (*n* = 10), and scented teas (*n* = 9). All the scented teas evaluated were teas flavored with different percentages of flowers, herbs or spices, ranging from 10% to 30%.

**Figure 3:**
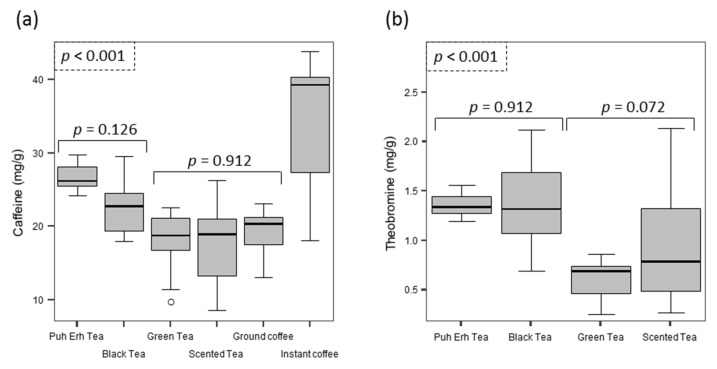
Distribution of the concentrations (in mg/g) of caffeine (**a**) and theobromine (**b**) detected in the different type of tea leaves and coffees evaluated.

Figure 3 shows the results obtained for caffeine and theobromine content distribution for tea samples. Theophylline was not included in the calculations as this compound was not detected in all tea samples and, when it was detected, levels were always below the quantification limit. For the two quantified compounds, there was a significant difference between the different types of tea evaluated (*p* < 0.001). When caffeine was measured, the post-hoc test indicated that the highest level was obtained for pu-erh and black teas (*p* = 0.126), whereas scented and green teas gave the lowest content. There were no significant differences in terms of caffeine content between green teas, scented teas and ground coffee (*p* = 0.912). In the case of theobromine, the post-hoc test showed that samples can be grouped into two groups: (i) pu-erh and black teas, which gave the highest theobromine contents (*p* = 0.981) and (ii) scented and green teas (*p* = 0.072).

### 3.4. Coffees

Twenty-three samples of commercial brands of ground coffee and 10 samples of instant (soluble) coffees were evaluated (Figure 3). In the case of instant coffees, eight samples corresponded to regular coffees and a mean caffeine content of 35.7 mg/g (sd = 6.9, ranging from 18.0 to 43.8 mg/g) was obtained. The other two samples were decaffeinated instant coffees and caffeine contents were 1.6 and 2.8 mg/g.

**Figure 4:**
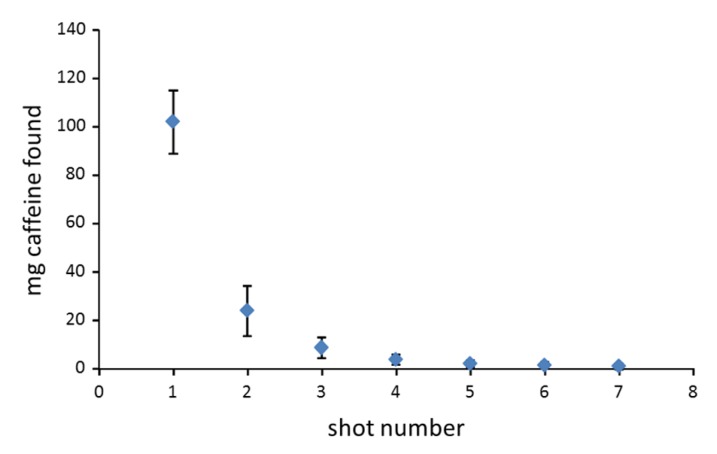
Evolution of the mg of caffeine detected in seven consecutive espresso shots collected using the same charge of ground coffee (*n* = 4, verticals bars show the calculated standard deviation).

Mean caffeine content in ground coffees was 19.3 mg/g (sd = 2.7, ranging from 13.0 to 23.1 mg/g), which was found to be equivalent to the caffeine levels in green and scented teas (*p* = 0.912), and significantly lower than levels in pu-erh and black teas. The amount of caffeine in a coffee beverage is not the same as the dry weight content since less than 100% of the total caffeine of the powder is usually extracted into the beverage. The caffeine content in coffees varies largely and depends on several factors, such as the type and amount of ground coffee used, the volume of hot water used for the extraction, the extraction time, and the temperature. In Spain, one of the most typical ways to drink a coffee is as an espresso shot, using high pressure and low contact time, 20–30 s, to create small cups (usually ranging from 30 to 60 mL) of an intensely flavored coffee. To evaluate the real amount of caffeine extracted in conventional espressos served in standard coffee-bars, some samples of ground coffee and espresso shots were obtained from four premises. To determine the total amount of caffeine present in each sample, the average weight of ground coffee applied in each of the premises for the preparation of espresso shots was measured, which ranged from 6 to 8.5 g, and a sample of ground coffee was analyzed to determine the dry weight content of caffeine. In each of the premises, seven consecutive shots were collected using the same charge of ground coffee in separate recipients. Figure 4 shows the mean amount (in mg) of caffeine found for the four premises at the different shots evaluated. The amount of caffeine obtained from the first shot ranged from 88 to 116 mg and the volume of this shot ranged from 30 mL to 45 mL. The percentage of caffeine extracted in this first shot ranged from 64% to 76% of the total caffeine present in the ground coffee sample. Amounts of caffeine obtained in the second shots ranged from 14 mg to 36 mg (10% to 20%). There was an exponential decrease in the amount of caffeine found in every shot and >90% of the total caffeine was extracted within the first three shots.

## 4. Discussion

### 4.1. Beverages and Chocolate Drinks

Soft drinks, including energy drinks, were the samples that gave the lowest variability (relative standard deviation, RSD, <8% despite samples being obtained from different batches). This fact and the high similarity in the content of caffeine for the different brands evaluated indicates that caffeine was artificially added to the samples in all of the cola and energy drinks evaluated, and that kola nut extracts were not used. All the conventional soft drinks and energy drinks evaluated presented caffeine levels that were equivalent to the values declared by the companies. It was observed that all diet-cola soft drinks evaluated had around 25% more caffeine than regular colas.

Bottled or canned teas presented a larger heterogeneity (RSD values ranged from 8.2% to 12.6%), presumably due to the caffeine and theobromine in these beverages being of natural origin from the tea leaves [43]. The mean caffeine concentrations found in the different brands of bottled teas evaluated gave significant differences (*p* < 0.001, Figure 1c), with mean caffeine levels of 43.5 and 55.5 mg/L for the two black iced teas evaluated and 35.3 mg/L for the green tea beverage. These results indicate that commercial bottled teas have between 2–3 times less caffeine content than cola soft drinks.

Chocolate drinks showed the lowest caffeine concentration (mean = 18.4 mg/L) of all the beverages evaluated. As in the case of bottled teas, the source of caffeine for these samples is of natural origin (cocoa beans), which also leads to a large variability between samples (RSD = 29.3%). Despite the fact that the methylxanthine present at the highest concentration in cocoa is theobromine (around 10 times more than caffeine in the samples evaluated), this compound produces very minor subjective effects in comparison with caffeine [44]. Therefore, taking into account only caffeine concentrations, chocolate drinks are the beverages that would be expected to present the least psychostimulant activity.

### 4.2. Foods

All the solid foods evaluated presented a large variability in the methylxanthine content due to their natural origin. The results indicate that the product with the highest dry weight content of caffeine was instant coffee with a mean percentage of 36 mg·g^−1^ (ranging from 18 to 44 mg·g^−1^, Figure 3), which agrees with previous studies where mean caffeine content in instant coffees of 33 mg·g^−1^ (ranging from 16 to 44 mg·g^−1^) were obtained [45,46,47]. The high dry content of caffeine in instant coffees is the result of the use of coffee beans with a large proportion of caffeine (i.e., *robusta* variety) for its production and that after a pressurized extraction step with water at very high temperatures (up to 200 °C) to obtain a coffee extract with around 15% solids, the brewed coffee is filtered and concentrated to a solid content of 25–70% [48]. After dehydration, a dry form is obtained with higher caffeine content than the starting ground coffee. In the case of decaffeinated instant coffees, the caffeine percentages obtained (1.6 and 2.8 mg·g^−1^) conform to EU legislation [49], which requires decaffeinated instant coffee to have a caffeine content not exceeding 3 mg·g^−1^.

Despite instant coffees presenting the highest amount of caffeine by dry weight, and even though a 100% of caffeine is extracted with hot water from this powder, the conventional amount of product used for preparation of a standard instant coffee is only 2 g of powder (as sold in individual sticks). This means that the amounts of caffeine in instant coffee beverages may range from 36 to 87 mg.

There are two main types of varietals for coffee beans, *Coffea arabica* and *Coffea canephora* (also known as *robusta* coffee). The caffeine content differs between the two varieties: in *robusta*, beans usually range from 1.7% to 4.0% (*w*/*w*) whereas in *arabica*, they range from 0.8% to 1.4% [48]. As the International Coffee Organization indicates [50], the price of *arabica* beans is normally about 40–50% higher than *robusta* beans. Due to the more attractive prices, most standard supermarket coffees and practically all instant coffees are exclusively or mainly *robusta* and it is necessary to go to specialty stores or to purchase more expensive brands to obtain 100% *arabica* coffees. The results obtained in the different brands evaluated gave caffeine percentages ranging from 1.3% to 2.6%. Only two of the samples evaluated were clearly indicated by the manufacturers to be 100% *arabica*, and these yielded the lowest percentages of caffeine (1.3% and 1.4%). All other ground coffees (*n* = 21) gave caffeine percentages ≥1.6%, which are mainly in the range expected for *robusta* variety or blends with high percentages of *robusta*. These results confirm that standard ground coffees sold in the supermarkets evaluated are mainly composed of the *robusta* variety.

When tea leaves were compared with ground coffee, it was found that pu-erh (*p* < 0.001) and black tea leaves (*p* = 0.006) contained higher caffeine contents than ground coffees (Figure 3), whereas ground coffee and green and scented tea leaves gave equivalent levels (*p* = 0.912). The higher caffeine content by dry weight of some types of tea leaves when compared to ground coffee was also observed in previous studies [45].

The amount of methylxanthines ingested from coffees and teas is not the total dry weight amount as these products are consumed in the form of an aqueous extract and usually less than 100% of the total methylxanthines is extracted, except in the case of instant coffees. In the present study, espresso coffees have been studied as this is the most conventional way of preparing coffee beverages in Spain. The percentage of caffeine extracted in conventional espresso shots ranged from 64–76%, which yielded amounts of caffeine in every espresso ranging from 88 to 116 mg (Figure 4). This agrees with previous studies where median values between 73 and 140 mg caffeine/serving have been found for espresso coffees [47,51,52]. These results indicate that espresso coffees have a higher amount of caffeine than instant coffees and suggest that if no other caffeine beverage or food is consumed, 4–5 espresso coffees per day might be considered as safe given that this quantity provides an amount of caffeine that is below the 400 mg limit proposed by the EFSA [21].

The caffeine concentrations detected in espresso coffees were very high, 2800–3700 mg/L for 30 mL shots and 1500–1800 mg/L for 60 mL shots, in comparison with other beverages. However, this method of preparing coffee does not result in the coffee beverages with the highest total amount of caffeine. Drip brewed coffees always present lower caffeine concentrations than espressos but this is due to their large volume (e.g., standard “medium” size brewed coffees in the USA is 473 mL, 16 oz.). It has been reported that the amount of caffeine present in a cup of drip brewed coffee tends to be higher than in espresso shots, when similar amounts of ground coffee are used in both methods, as the drip brew method extracts caffeine more efficiently [53,54]. The USA National Coffee Association [55] has established a coffee-to-water “golden ratio” of about 30-60 mg of ground coffee for every mL of water (1-2 tablespoons for every 177 mL). This means that for a coffee with 2% of caffeine, a maximum of 568 mg of caffeine could be extracted for a “medium-sized” coffee. A study evaluating different commercial brands of 473 mL brewed coffees found that caffeine amounts present in these beverages ranged from 148 to 564 mg, which were 3–5 times higher than amounts found in espresso coffees served at the same premises (58 to 93 mg) [52].

In the case of tea leaves, the efficiency in extracting methylxanthines is usually >80% using hot water at temperatures ≥80 °C [39]. Although the dry weight content in tea leaves is equal or higher than in ground coffee, the amount of leaves used for the preparation of tea beverages (1.5–2.0 g) is smaller than the weight of ground coffee used for espresso shots (6–8.5 g). Therefore, a maximum of 44–60 mg of caffeine (assuming 100% extraction) can be obtained in the case of the pu-erh and black teas evaluated in the present study, and 33–45 mg for the green teas. Due to the different ways of preparing the aqueous extracts, tea beverages tend to present at least 2–3 times lower amounts of caffeine than espresso coffees, but the amount of caffeine present in black and pu-erh tea extracts is comparable to that of instant coffee beverages. In the case of green teas, the amount of caffeine in the aqueous extract is similar to the amount present in a conventional portion size of cola soft drinks (i.e., 330 mL).

In the case of chocolates, levels of methylxanthines vary in accordance with the percentage of cocoa in the samples evaluated. The content of theobromine was approximately 10 times higher than caffeine; these values agree with previous studies [32,33,56,57,58]. Chocolate is a suspension that melts at body temperature during its consumption, giving a smooth suspension of particulate solids in cocoa butter [59]. Different studies have indicated that chocolate and other cocoa-containing foods contribute small amounts (<1.5%) of caffeine to the diet, and that >98% of all caffeine consumed came from coffee, tea and soft drinks, including energy drinks, with coffee being the main source in people older than 18 years and soft drinks in children aged 2–17 years [15,36]. The mean chocolate intake in a prospective study of 20,951 people was found to be 4.6 g/day, which rose to 7.0 g/day when those who did not consume chocolate at all were excluded [60]. This amount is far less than the 360–520 g of dark chocolate (80–85% cocoa) or >1 kg of milk chocolate, calculated from the values obtained in the present study, that would be needed to reach the upper limit of 400 mg of caffeine suggested by the EFSA [21]. It has also been reported that chocolate doses of up to 100 g/day present beneficial effects lowering the risk of cardiovascular diseases and stroke [60].

## 5. Conclusions

The results obtained in the present study show some significant trends. In relation to their dry weight content, tea leaves have the same or higher caffeine content than ground coffee. However, the different ways of preparing the extract leads to caffeine contents in espresso coffees being at least 2–3 times higher than in teas. Although espresso coffees present a very high concentration of caffeine due to the small volume of the prepared beverage, the total amount of caffeine in espressos is lower than in other conventional methods of preparing coffee, such as drip brewed coffee, when using similar amounts of ground coffee in their preparation, because the extraction efficiency of espresso shots is limited (<76% caffeine extracted in a single shot).

When comparing the total amount of caffeine ingested for a single consumption of each one of the different products evaluated, it was observed that coffee and energy drinks present the highest caffeine content. The amount of caffeine obtained in the different espresso coffees evaluated ranged from 88 to 116 mg. The most popular energy drinks among young people in Europe have a caffeine concentration of 320 mg/L. Taking into account the two most common serving sizes of commercial energy drinks (250 and 330 mL), this means that the caffeine content for each beverage ranges from 80 to 106 mg, at the same level as an espresso coffee. Energy drinks are particularly important as their consumption has been increasing exponentially since their introduction at the end of the last century, especially among young people aged between 13 and 24 years [7,15]. Another problem associated with energy drinks is that recent studies among college students have shown that >35% of energy drink consumers declare that they increase the number of consumptions and combine the beverage with alcohol while partying [61,62], which seems to increase the rate of alcohol-related injury [7].

## Supplementary Materials

The following are available online at www.mdpi.com/2304-8158/6/12/109/s1.
