# Supplementary material for: Methylxanthine Content in Commonly Consumed Foods in Spain and Determination of Its Intake during Consumption
Source: Foods. 2017 Dec 4;6(12):109. doi: 10.3390/foods6120109 (PMC5742777; doi:10.3390/foods6120109)
Supplement: Supplementary file 1 [file foods-06-00109-s001.pdf]

SUPPLEMENTARY MATERIALS

Methylxanthine content in commonly consumed foods in Spain and determination of its intake during consumption

Juan M. Sanchez

Chemistry Dept., University of Girona, Girona, Spain

Sample treatment

All solid samples (chocolate, ground coffee and tea leaves) were finely grounded and oven dried at 110^{0}C for at least 3-5 h before being treated.

Chocolate foods

Official method 980.14 of the AOAC was applied with some modifications for chocolate foods [1]. One gram of a dry chocolate was accurately weighted, mixed with 25 ml of petroleum ether to extract the fat content, and centrifuged at 1500 rpm for 10 min. The liquid phase was eliminated. The extraction and centrifugation step was repeated two times for each sample. The final solid was allowed to air-dry overnight in a fume hood. Once dried, the solid was dissolved with around 80 ml Milli-Q hot water at 80-90^{0}C and maintained at this temperature for 30 min to ensure complete solubilization of the methylxanthines. When at room temperature, the final solution was filtered through a 45 µm RC filter and transferred to a 250 ml volumetric flask to obtain a known volume of sample solution before HPLC analysis.

Chocolate drinks

For chocolate drinks, a clean-up with solid-phase extraction was applied to remove lipophilic components in the beverages [2]. 2 ml of a chocolate drink were mixed with 20 ml of hot water at 80-90^{0}C and maintained at this temperature for 15 min. The final solution was transferred to a 25 ml volumetric flask filled with Milli-Q water. C18 solid-phase extraction (SPE) cartridges were conditioned with 2 ml of methanol and 2 ml of water. 1 ml of the 20 ml mixture of chocolate drink was passed through the conditioned SPE cartridge. Recovery of the retained compounds in the SPE cartridge was done with 3 ml of pure methanol. The methanol solution was concentrated to dryness under vacuum at 40^{0}C and the residue was dissolved in 1 ml of Milli-Q water and analyzed by HPLC.

Tea leaves

A two-step extraction with hot water was applied [3,4]. One gram of dried tea leaves was accurately weighted and mixed with around 40 ml of water at 90^{0}C, the mixture was shaken and the extraction took place over 15 minutes at 90^{0}C. The liquid solution was separated and a second extraction of the remaining tea leaves was done in the same conditions. The two

resulting liquid solutions were mixed in a predetermined volumetric flask, filled with milli·Q water. A portion of the resulting solution was filtered through a 45 µm filter and analyzed by HPLC.

Ground coffee and instant coffee

The same extraction procedure as for tea leaves with hot water at 90^0C was used for ground coffee but only one extraction was performed, as indicated by the international standard ISO 20481:2008 [5].

In the case of instant coffee, 0.5 grams of powder were dissolved with around 80 ml Milli-Q hot water at 80-90^0C and maintained at this temperature for 1-2 min. When at room temperature, the final solution was filtered and transferred to a 100 ml volumetric flask. This sample was diluted 10 times before HPLC analysis.

HPLC method development and quality parameters

Despite some studies having proposed using isocratic HPLC separations [6-9], the use of gradient elution is usually required for an adequate separation of methylxanthines and interferences in a reasonable time in complex matrices such as tea leave extracts [9-16]. The evaluation of different isocratic elution conditions with the C18 column used in this study showed that a methanol content of ≥30% is not adequate since the chromatographic resolution (R_s) between theophylline and caffeine was too small to ensure an adequate separation of the two compounds (R_s=0.98 and 1.11 at 40% and 30% MeOH respectively). Moreover, the capacity factor (k) was <0.75 for the first eluting compound, theobromine. On the other hand, the peak width at the base (w_b) for the last eluting compound, caffeine, was >2 min and >4 min for isocratic elution with 20% and 10% MeOH respectively. When analyzing complex matrices, such as tea leaf extracts, it was not possible to separate all three methylxanthines from other matrix components (mainly polyphenols, especially catechins) in any isocratic condition. These results confirm that gradient elution is required with octadecyl stationary phases for this application. The gradient conditions indicated in the experimental section allowed an adequate separation of the three methylxanthines and other matrix components in the extracts of green tea leaves (Figure S1). This type of tea was chosen for evaluation of the gradient conditions as levels of interfering catechins are higher in green teas

than in other teas because the fermentation processes during the tea manufacturing reduce their levels significantly [12].

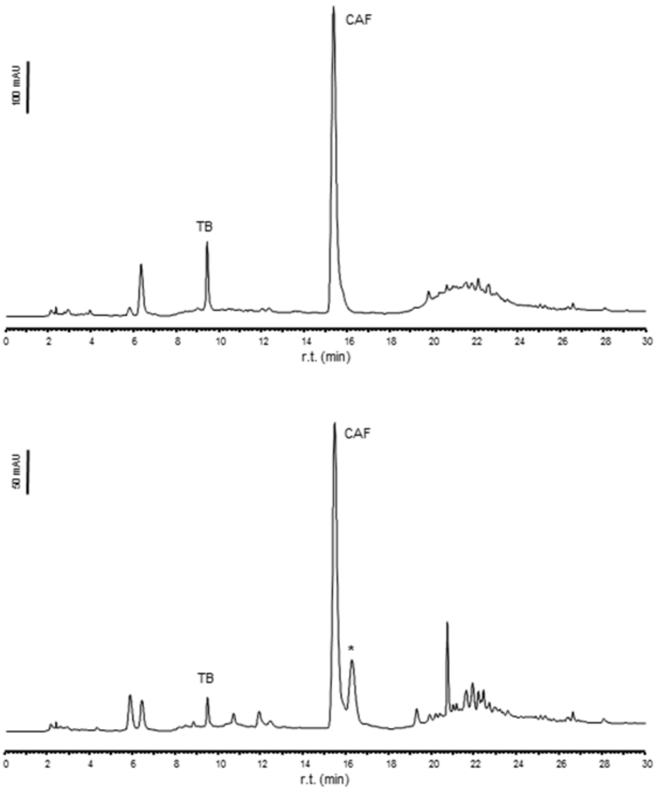

Figure S1. Chromatograms obtained in the analysis of a black tea (upper chromatogram) and a green tea (lower chromatogram). TB: theobromine, CAF: caffeine, *catechin compound.

The stability of the chromatographic column was determined by evaluating the retention times of standards and samples injected over a one-month period. Residual standard deviations (RSD) of <0.8% for the intra-day and <2.1% for the inter-day retention times of the three methylxanthines were obtained. Moreover, no significant differences were obtained between

the slopes of the different calibration curves (n=15, prepared by different laboratory technicians on different days), with variations in the slopes of <4.6%. Linearity was confirmed for all target compounds from the evaluation of the distribution of residuals and the application of the lack-of-fit F test (n=6, three replicates at each level). Determination coefficients (R^2) were always >0.997.

Determination of the instrumental detection limit (LODs) was based on the standard deviation (sd) of a reagent blank and the slope measured [17]. A standard mixture containing around 0.5 mg/l for each methylxanthine was used as a reagent blank and analyzed under repeatability conditions (n=10). The sd obtained was taken as the sd of the blank (sd_{bl}) and the $3 \cdot sd_{bl}$ criterion was applied to calculate LODs and the $10 \cdot sd_{bl}$ criterion for limits of quantification (LOQs). LOQs were set at 0.7 mg/l and LODs at 0.2 mg/l. Blank analysis always gave signals below the calculated LODs when this procedure was applied.

A soft drink, containing only caffeine, and a chocolate sample were used for the evaluation of the precision. A two-factor fully-nested design [18-20] with four replicates a day for a period of 3 days was used. Repeatability of caffeine determination in the soft drink sample was 0.9% and intermediate precision was 1.1%. The chocolate sample gave a repeatability value of 4.8% for caffeine (low level) and 3.7% for theobromine (high level), whereas intermediate precision was 5.2% for caffeine and 4.2% for theobromine.

To assess the trueness of the methodologies used, replicates (n=3) for different types of samples evaluated (tea leaves, ground coffee, chocolates, instant coffee and soda drinks) were obtained. Each replicate was divided into two equal portions and one was fortified with the three methylxanthine compounds. Duplicates were treated according to the type of sample and analyzed to obtain the recoveries. For beverages samples the recoveries ranged from 95 to 103%. In the case of solid samples, recoveries ranged from 86 to 112%.

Quality controls

Before starting a sequence of samples, a standard was analyzed sequentially until stable retention times were obtained for the analytes (<0.1 min variation in retention times in three consecutive analyses). Thereafter, a method blank was injected to confirm that no system contamination took place. Calibration was performed using six standards. A blank and a quality control were analyzed between each series of 5 samples.

Bibliography

1. AOAC Official Method 980.14. "Theobromine and caffeine in cacao products". **1981**
2. Carlin-Sinclair, A.; Marc, I.; Menguy, L.; Prim. D. The determination of methylxanthines in chocolate and cocoa by different separation techniques: HPLC, instrumental TLC, and MECC. *J Chem Edu* **2009**, 86, 1307-1310
3. Khokhar, S.; Magnusdottir, S.G.M. Total phenol, catechin, and caffeine contents of teas commonly consumed in the United Kingdom. *J Agric Food Chem* **2002**, 50, 565-570
4. Perva-Uzunalic, A.; Skerget, M.; Knez, Z.; Weinreich, B.; Otto, F.; Grüner, S. Extraction of active ingredients from green tea (Camellia sinensis): Extraction efficiency of major catechins and caffeine. *Food Chem* **2006**, 96, 597-605
5. ISO 20481:2008. Coffee and coffee products – Determination of caffeine content using high performance liquid chromatography (HPLC) – Reference method. Technical Committee ISO/TC 34, Food Products, Subcommittee SC 15, Coffee. **2011**
6. Chuo, K.H.; Bell, L.N. Caffeine content of prepackaged national-brand and private-label carbonated beverages. *J Food Sci* **2007**, 72, C337-C342
7. Wang, X.; Hu, S.; Wan, X.; Pan C. Effect of microbial fermentation on caffeine content of tea leaves. *J Agric Food Chem* **2005**, 53, 7238-7242
8. De Camargo, M.C.R.; Toledo, M.C.F. HPLC determination of caffeine in tea, chocolate products and carbonated beverages. *J Sci Food Agric* **1999**, 79, 1861-1864
9. Srdjenovic, B.; Djordjevic-Milic, V.; Grujic, N.; Injac, R.; Lepojevic, Z. Simultaneous HPLC Determination of Caffeine, Theobromine, and Theophylline in Food, Drinks, and Herbal Products. *J Chromatogr Sci* **2008**, 46, 144-149
10. Fernández, P.L.; Martín, M.J.; González, A.G.; Pablos, F. HPLC determination of catechins and caffeine in tea. Differentiation of green, black and instant teas. *Analyst* **2000**, 125, 421-425
11. Khokhar, S.; Magnusdottir, S.G.M. Total phenol, catechin, and caffeine contents of teas commonly consumed in the United Kingdom. *J Agric Food Chem* **2002**, 50, 565-570
12. Zuo, Y.; Chen, H.; Deng, Y. Simultaneous determination of catechins, caffeine and gallic acids in green, Oolong, black and pu-erh teas using HPLC with a photodiode array detector. *Talanta* **2002**, 57, 307-316
13. Friedman, M.; Levin, C.E.; Choi, S.H.; Kozukue, E.; Kozukue, N. HPLC analysis of catechins, theaflavins, and alkaloids in commercial tea and green tea dietary supplements: comparison of water and 80% ethanol/water extracts. *J Food Sci* **2006**, 71, C328-C337
14. Crozier, T.W.M.; Stalmach, A.; Lean, M.E.J.; Crozier, A. Espresso coffees, caffeine and chlorogenic acid intake: potential health implications. *Food Funct* **2012**, 3, 30-33

15. Lee, L.S.; Kim, S.H.; Kim, Y.B.; Kim, Y.C. Quantitative analysis of major constituents in green tea with different plucking periods and their antioxidant activity. *Molecules* **2014**, 19, 9173-9186
16. Alañón, M.E.; Castle, S.M.; Siswanto, P.J.; Cifuentes-Gómez, T.; Spencer, J.P.E. Assessment of flavanol stereoisomers and caffeine and theobromine content in commercial chocolates. *Food Chem* **2016**, 208, 177–184
17. LGC. EURACHEM Guide: The fitness for purpose of analytical methods. LGC: Middlesex, UK, **1998**
18. ISO 5725-3. Accuracy (trueness and precision) of measurement methods and results – Part 3: intermediate measures of the precision of a standard measurement method. ISO: Geneva, Switzerland, **1994**.
19. Maroto, A.; Riu, J.; Boqué, R.; Rius, F.X. Estimating uncertainties of analytical results using information from the validation process. *Anal Chim Acta* **1999**, 391, 173-185
20. González, A.G.; Herrador, M.A.; Asuero, A.G. Intra-laboratory assessment of method accuracy (trueness and precision) by using validation standards. *Talanta* **2010**, 82, 1995-1998
